# Bet on NETs! Or on How to Translate Basic Science into Clinical Practice

**DOI:** 10.3389/fimmu.2016.00417

**Published:** 2016-10-14

**Authors:** Giuseppe A. Ramirez, Angelo A. Manfredi, Patrizia Rovere-Querini, Norma Maugeri

**Affiliations:** ^1^Unit of Internal Medicine and Immunology, IRCCS Ospedale San Raffaele, Milano, Italy; ^2^Università Vita-Salute San Raffaele, Milano, Italy; ^3^Division of Immunology, Transplantation and Infectious Diseases, IRCCS Ospedale San Raffaele, Milano, Italy

**Keywords:** neutrophil extracellular traps, autoimmune diseases, clinical practice, diagnostic techniques, therapy, assays

Neutrophil extracellular traps (NETs) comprise neutrophil DNA, citrullinated (deiminated) histones, and proteases (1, 2). NET formation *in vivo* mostly occurs at the sites of inflammation. Neutrophils, adhering to the endothelium or after extravasation, generate NETs upon interaction with microbes, activated platelets, cytokines, alarmins, such as high-mobility group box 1 (HMGB1), or uric acid. Environmental cues, such as hyperosmolarity or hyperglycemia, neurotransmitters, and some autoantibodies also trigger NET generation (3–7). *In vitro*, two main pathways are activated: suicidal and vital NETosis (8). In the former, membrane integrity is disrupted and neutrophils die. In contrast, after “vital” NETosis neutrophils still migrate, chase bacteria, and extrude residual nuclear material through exocytosis (9). Generation of reactive oxygen species (ROS) and fusion of neutrophil primary granules with the nuclear membrane that promote interactions between elastase, myeloperoxidase (MPO), and DNA are features of suicidal NETosis. Their role in vital NETosis is debated (8, 10–12). Activation of the autophagic pathway is intermingled with NET generation. Autophagy sustains the metabolic requirements of the extensive intracellular vesicular formation, transport, and fusion associated with NET generation. It also sustains neutrophil survival (5).

Neutrophils undergo extensive *ex vivo* manipulations and methodological approaches vary among research groups, possibly explaining some discrepancies and nurturing some healthy skepticism (13). Some reports indicate a role of the mitochondrial DNA, which is devoid of histones, in NET generation. The relative contribution of nuclear vs. mitochondrial DNA to extracellular traps generation remains a controversial issue (14).

Under physiological conditions, NETs enhance the host response to microbes by (i) providing a template concentrating humoral innate immune molecules, such as pentraxin 3 (PTX3) and complement, together with microbicidal molecules, such as histones, MPO, proteinase 3 (PR3), or cathelicidin, and (ii) contrasting the hematogenic spread of pathogens through immunothrombosis (i.e., activation of platelets and of the coagulation cascade through NET components) (15). The actions of NET-embedded von Willebrand factor, of citrullinated histones, and of negative charges on platelet recruitment/activation and on the progression of the coagulation cascade contribute to thrombosis. Excellent reviews on this issue have been published (16, 17). The bioactive molecules integrated within the chromatin threads may vary according to the inciting stimuli or the environment. The characterization of the NETs proteome is a fascinating challenge for the near future.

Neutrophils are abundant in the blood, and their concentration and activation state increase following surges of systemic cytokines during acute phase responses. Thus, NETs are readily available and easily renewable tools for the first-line response to infectious agents. However, there is a price to pay. During acute infections (e.g., during sepsis), NETs favor progression toward septic shock, disseminated intravascular coagulation, and acute respiratory distress syndrome (ARDS), as well as tissue damage and organ failure, possibly because of the deleterious effects of immunothrombosis. On the other hand, the imbalance between the rate formation and degradation of NETs might favor the persistence and the presentation of autoantigens and facilitate autoimmunity (1).

Systemic lupus erythematosus (SLE) is a paradigm of the pathogenic *continuum* that links nuclear antigens exposure to autoimmunity. Neutrophil from patients with SLE show prolonged survival (possibly through enhanced autophagy) and are thus endowed with additional chances to generate NETs. In addition, the clearance of extracellular DNA in SLE is impaired either as a genetically determined trait influencing waste disposal or due to antibodies toward regulatory molecules, such as DNases (18, 19).

Antineutrophil cytoplasmic antibodies (ANCA) represent a variant on the theme of autoantibodies elicited following enhanced generation and/or persistence of NETs. The formation of NETs at sites of vascular inflammation in ANCA-associated vasculitides (AAV) and the ability of ANCA to perpetuate the generation of NETs were described in 2009 (3). Later on, dendritic cells have been found to capture ANCA targets (i.e., MPO and PR3) from NETs and to present them to T lymphocytes, while the susceptibility to ANCA generation was found to be deeply rooted in genetics. Neutrophil priming with HMGB1, a well-established inducer of NETs and of autophagy, contributes to the action of ANCA (20).

In rheumatoid arthritis, NETting neutrophils infiltrate the synovium and rheumatoid nodules, while anti-citrullinated peptides antibodies apparently boost the generation of NETs (21). B cells that expand and differentiate within ectopic synovial germinal centers frequently target deiminated antigens generated during NETosis (22, 23) and/or as a consequence of the release of bioactive peptidylarginine deiminases in the synovial fluid.

Anti-phospholipid antibodies in combination with ROS and platelet-assisted TLR4 stimulation induce neutrophils to form NETs, which in turn precipitate intravascular thrombosis (15). Conversely, NET degradation appears to be impaired in a fraction of patients with anti-phospholipid syndrome (19). Neutrophils infiltrate the pancreas of patients with type 1 diabetes mellitus (T1DM) (24) and, according to experimental models and indirect clinical evidence (systemic levels of NETs by-products in serum), cause an IFNα response, autoantibody generation, and β-cells destruction through *in situ* NETosis (25, 26).

## Indicators and Markers of NETosis

The notion that NETs contribute to a wide range of autoimmune diseases so far has had little impact on the clinical practice. Reasons include

(i) the low threshold of activation of neutrophils, which limits the development of robust, easy-to-perform, and cheap diagnostic assays;
(ii) high costs of clinical trials; and
(iii) the lack of insight on appropriate targets to safely target NETosis.

Three main approaches are currently used in research laboratories:

- analytical assays based on fluorimetry for cell-free DNA or on ELISAs for soluble NET by-products (DNA–MPO or DNA–neutrophil elastase complexes, citrullinated histones) (3, 5);
- confocal microscopy for neutrophil enzymes along extracellular DNA lattices (3, 5); and
- flow cytometry, based on nuclear morphology and variations in MPO distribution (11) or staining of citrullinated histones or DNA (27).

**Figure 1:**
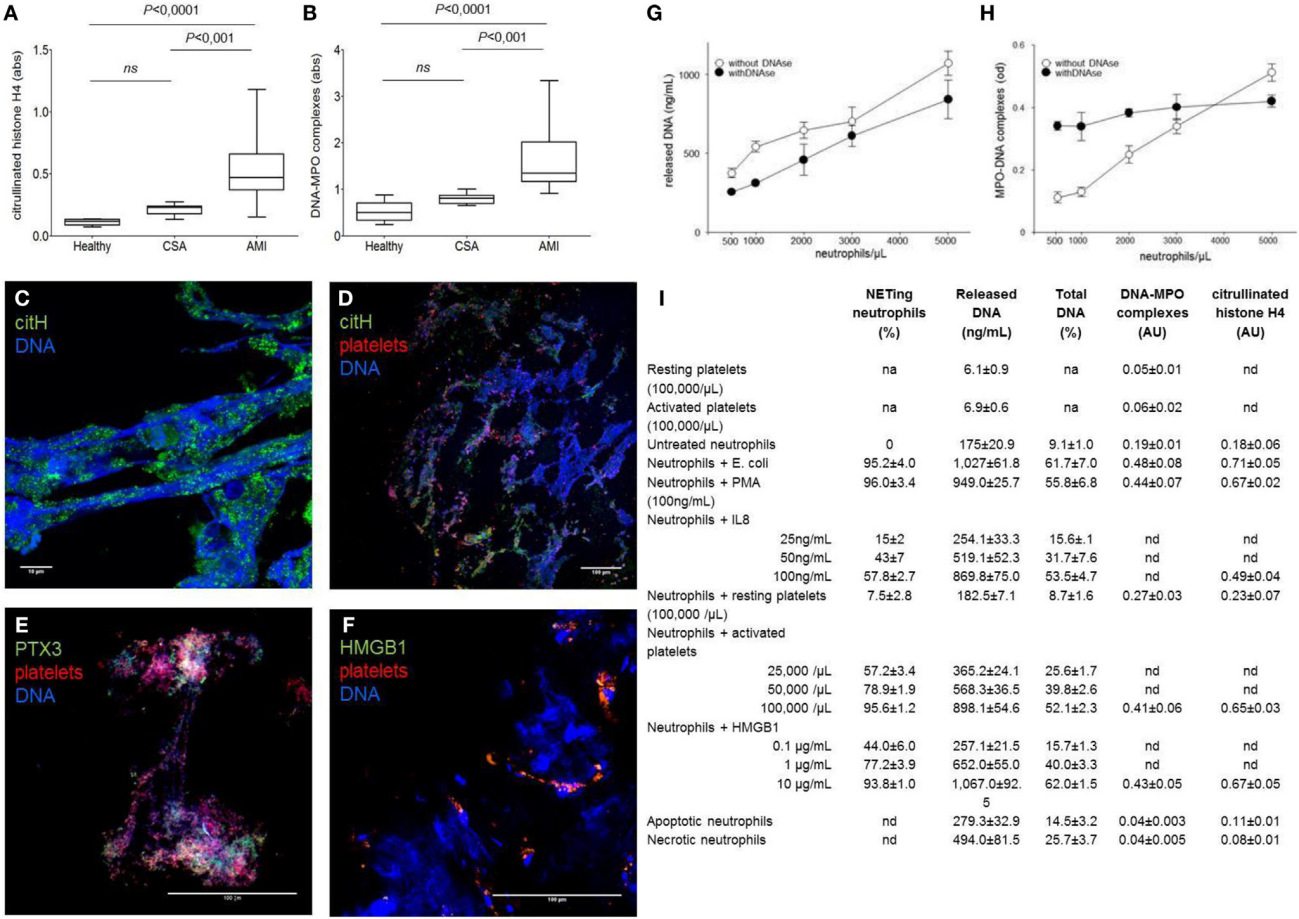
**NETs by-products are easily detected in the plasma of patients ongoing acute myocardial infarction and reflect the NETs that characterized coronary thrombi**. **(A–F)** Coronary thrombi and plasma samples were obtained from 26 consecutive patients with STEMI underwent percutaneous intervention within 1–6 h from the onset of chest pain. NETs by-products were determined in plasma samples (anticoagulated with EDTA, venipuncture through a 19-gage butterfly needle) and processed within 15 min of sampling. Whole blood was centrifuged 15 min (320 *g*; 4°C), and retrieved plasma was further centrifuged (5 min at 100,000 *g*, 4°C) to eliminate any cellular debris. Obtained plasma was then aliquoted and frozen until de-determination of NETs by-products. All steps of samples preparation were performed in sterile conditions. **(A,B)** The amount of DNA–MPO complexes and the quantification of histone H4 citrullinated were assessed in the plasma samples of the same AMI patients in which were analyzed the composition of coronary thrombi, patients with chronic stable angina (CSA) candidates for coronary revascularization, and sex- and age-matched healthy donors. **(C–F)** Representative confocal analysis of coronary thrombi of AMI patients obtained during revascularization procedure. NETs were identified as DNA lattices with evident citrullination of histones **(C,D)** and by the presence of specific neutrophil proteins such as PTX3 **(E)**. We consistently detected the presence of NETs in close proximity of platelets **(D–F)** that frequently express HMGB1 **(F)** [adapted from Maugeri et al. (5)]. **(G,H)** *The use of DNAs impaired the sensitivity on NET determination*: addition of DNase seems useful when low numbers of neutrophils are used. Freshly purified human neutrophils (0.5–5 × 10^6^) of healthy donors were placed on poly-l-lysine-coated slides for 20 min at 37°C, then were challenged with PMA (100 ng/mL) for 20 min at 37°C. Then, treated or not with DNase I, after incubation plates were centrifuged 5 min (320 *g*; 4°C), supernatants were retrieved and further cleared (5 min at 100,000 *g*) and frozen until determination of NETs by-products and cell-free DNA as we previously described [adapted from Maugeri et al. (5)]. **(I)** *Quantitative assays for NET formation*. The results of a series of independent experiments carried out with cells of different donors are presented in this back-to-back comparison of different quantitative assays to estimate the degree of NET generation. Neutrophils were stimulated with a large panel of agonists to reproduce the environmental variability that occurs under physiological conditions. Quantitation of cell-free DNA is consistent with the degree of NET generation as estimated by direct microscopy visualization and MPO/DNA- or citrullinated histone-based techniques in this set of experiments, where purified neutrophils were employed [adapted from Maugeri et al. (5)].

Fluorimetry is not time-consuming and generate semi-quantitative information that can be associated with clinical variables. However, it does not unambiguously identify the source of DNA (neutrophils or other cells) or the process by which DNA was released (NETosis, necrosis, necroptosis, etc.). The association of DNA fragments to neutrophil enzymes and the citrullination of histones are relatively specific for NETosis. Thus, determination of MPO–DNA complexes and/or of citrullinated histones selectively reveals the amount of NET by-products in biological fluids. Human studies monitoring *in vivo* NETs formation (3, 5) also revealed a concordant rise in NETs by-products in plasma, suggesting that ELISA is sufficient *per se* to assess the degree of NET formation in human inflammatory diseases (Figures 1A–F). Furthermore, parallel quantitation of *in vitro*-generated NETs by confocal microscopy and concomitant measurement of DNA, DNA–MPO complexes, and citrullinated histones in cell-free supernatants drives to concordant results (Figures 1G–I). Thus, we believe that, at present, only ELISA assays on plasma samples meet the requirements of robustness, reproducibility, and easiness for widespread clinical use.

However, it does not discriminate between vital or suicidal NETosis nor assess the relative contribution of NET generation and catabolism. Standardized protocols will be necessary for transfer these assays to the clinics (28). For example, DNases for sample enrichment influence the sensitivity of MPO/DNA-based analytical assays (Figures 1G,H).

Microscopy allows a direct visualization of NETs. In expert hands, it remains a powerful and informative tool, even more when combined with the determination of NET by-products (Figures 1A–F) (3, 5) and with high-throughput proteomic assays. However, due to the high inter-observer variability, it is not routinely used in clinical practice. Novel semi-automated image analysis techniques might circumvent this limit. Flow cytometry yields accurate and reproducible data. Large trials are needed to pave the way to their widespread use (11, 28). Besides improvements in NET detection, caution in the pre-analytical sample handling is mandatory, since neutrophils and platelets respond to physical and chemical stimuli during blood sampling and transportation. Activated platelets, in particular, release various signals – both soluble and associated with microparticles – that impact on neutrophil functions, including NET generation (12, 29).

## NETs, Drug Development, and Repositioning

Citrullination of histone residues is a key step in NETosis. Pharmacological or genetic tools to inhibit deiminating enzymes reduce the formation of NETs and their detrimental consequences in preclinical models (30–32). The actual selectivity and potential safety risks of the available pharmacological inhibitors are not yet established.

Mitochondrial generation of ROS triggers NET formation. In addition, oxidized mitochondrial DNA within NETs could contribute to its immunogenic potential. Interference with the respiratory chain and/or ROS scavenging exerts anti-inflammatory effects and clinical benefit in mice models of sepsis and SLE (33).

Targeting platelet/neutrophil reciprocal activation and platelet microparticle-associated moieties, HMGB1 in particular (5, 12, 34), and finding strategies aimed at restoring the phagocytosis of apoptotic substrates by neutrophils can exert a calming influence over NET generation (35) and thus appear promising. Other anti-NET treatments could aim at restoring and/or potentiating the NET clearance. NET-mediated lung injury in cystic fibrosis abates in response to nebulized DNase supplementation. Studies improving the drug delivery could pave the way for the application of this anti-NETs treatment to inflamed joints, kidney, lung, or skin in the setting of autoimmune diseases.

The repositioning of known drugs and agents has advantages over the development of new drugs, since toxicity/safety profiles are usually known and cost and time to bring agents to market abate. A proof-of-concept trial links reduced SLE flares to the metformin inhibition of mtDNA-enriched NETs. An action of metformin on HMGB1 release (5, 33) could also be involved. Heparins are being used in a wide range of diseases for effects independent of their anticoagulant properties (36), including conditions, in which NETs generation possibly plays a role. Heparins might interfere with the metabolic needs for NET generation since they restrict the activation of the autophagic flux (29). Prophylactic doses of low-molecular weight heparins, which are routinely used for thromboprophylaxis and for the prevention of pregnancy complications, indeed interfere with autophagy induction of neutrophils of healthy subjects and virtually abrogate the ability to generate NETs in response to various stimuli (29). Thus, interference with NETosis might be involved in the benefit of treatments selected mainly on empirical basis. Unfractionated heparin has also been shown to dismantle NETs after they have been generated, by liberating histones from the DNA backbone and destabilizing the chromatin threads (37).

In conclusion, accumulating data on the role of NETs in autoimmune diseases and in highly prevalent inflammatory conditions such as sepsis demonstrate that novel bioindicators and treatments might readily become available and improve the quality of patients care from tomorrow. Its time to take the next step.

## Author Contributions

GR, AM, PR-Q, and NM selected the bibliography, discussed the opinion approach, and wrote the manuscript.

## Conflict of Interest Statement

The authors declare that the research was conducted in the absence of any commercial or financial relationships that could be construed as a potential conflict of interest.
